# Post-Exercise Carbohydrate-Energy Replacement Attenuates Insulin Sensitivity and Glucose Tolerance the Following Morning in Healthy Adults

**DOI:** 10.3390/nu10020123

**Published:** 2018-01-25

**Authors:** Harry L. Taylor, Ching-Lin Wu, Yung-Chih Chen, Pin-Ging Wang, Javier T. Gonzalez, James A. Betts

**Affiliations:** 1 Department for Health, University of Bath, Bath BA2 7AY, UK; taylor210@googlemail.com (H.L.T.); y.chen2@bath.ac.uk (Y.-C.C.); 2 Graduate Institute of Sports and Health Management, National Chung Hsing University, Taichung 402, Taiwan; wuchinglin@icloud.com (C.-L.W.); pspgw@dragon.nchu.edu.tw (P.-G.W.)

**Keywords:** insulin sensitivity, exercise, carbohydrate metabolism, oral glucose tolerance test

## Abstract

The carbohydrate deficit induced by exercise is thought to play a key role in increased post-exercise insulin action. However, the effects of replacing carbohydrate utilized during exercise on postprandial glycaemia and insulin sensitivity are yet to be determined. This study therefore isolated the extent to which the insulin-sensitizing effects of exercise are dependent on the carbohydrate deficit induced by exercise, relative to other exercise-mediated mechanisms. Fourteen healthy adults performed a 90-min run at 70% $\dot{V}O_{2}$max starting at 1600–1700 h before ingesting either a non-caloric artificially-sweetened placebo solution (CHO-DEFICIT) or a 15% carbohydrate solution (CHO-REPLACE; 221.4 ± 59.3 g maltodextrin) to precisely replace the measured quantity of carbohydrate oxidized during exercise. The alternate treatment was then applied one week later in a randomized, placebo-controlled, and double-blinded crossover design. A standardized low-carbohydrate evening meal was consumed in both trials before overnight recovery ahead of a two-hour oral glucose tolerance test (OGTT) the following morning to assess glycemic and insulinemic responses to feeding. Compared to the CHO-DEFICIT condition, CHO-REPLACE increased the incremental area under the plasma glucose curve by a mean difference of 68 mmol·L^−1^ (95% CI: 4 to 132 mmol·L^−1^; *p* = 0.040) and decreased the Matsuda insulin sensitivity index by a mean difference of −2 au (95% CI: −1 to −3 au; *p* = 0.001). This is the first study to demonstrate that post-exercise feeding to replaceme the carbohydrate expended during exercise can attenuate glucose tolerance and insulin sensitivity the following morning. The mechanism through which exercise improves insulin sensitivity is therefore (at least in part) dependent on carbohydrate availability and so the day-to-day metabolic health benefits of exercise might be best attained by maintaining a carbohydrate deficit overnight.

## 1. Introduction

Physical activity is a powerful tool to improve insulin sensitivity and glycemic control [1]. Accordingly, increasing physical activity is a crucial counter-measure in reducing T2D prevalence [2,3]. On an acute level, single bouts of exercise consistently enhance insulin sensitivity and muscle glucose uptake in both insulin-resistant [4] and healthy individuals [5], although the acute effects of exercise on glucose *tolerance* are less clear [6,7].

Metabolic flexibility is a key aspect of insulin sensitivity and reflects the ability to switch between substrate sources for oxidation according to availability. Individuals with robust metabolic flexibility display high rates of fat oxidation in the fasted state, switching to high rates of carbohydrate oxidation in the fed (or insulin-stimulated) state. It has been suggested that impaired metabolic flexibility may be an early cause of insulin resistance via lipid accumulation [8,9], although the direction of the relationship between metabolic flexibility and insulin sensitivity remains unclear. Notwithstanding this, substrate selection in the fasted and fed state may be an important mechanism by which exercise alters insulin sensitivity and postprandial glycemia.

The beneficial effects of exercise on glycemic control and insulin sensitivity are thought to be mediated, at least partly, by whole-body carbohydrate status. The increased insulin action after a single bout of exercise, or during the early phases (6 d) of moderate-intensity exercise training (walking), are attenuated when the energy and/or carbohydrate utilized by exercise is replaced by dietary intake [10,11,12]. These responses appear to be largely driven by carbohydrate status rather than energy status, since an exercise-induced carbohydrate deficit increases insulin sensitivity even in the presence of energy balance, whereas an exercise-induced energy deficit in the presence of high muscle glycogen does not [13]. Furthermore, in rodents, adrenaline-induced muscle glycogen depletion enhances insulin sensitivity of the muscle to glucose transport [14], highlighting the role of muscle carbohydrate status in insulin sensitivity, independent of exercise.

Whilst a number of studies have assessed post-exercise glucose metabolism and/or insulin action under conditions of carbohydrate and/or energy replacement, these studies have all employed intravenous tests of glucose metabolism and insulin sensitivity. Notwithstanding the strengths of tightly-controlled intravenous tests, there is a need to understand postprandial responses with the oral ingestion of glucose. This is particularly important since the acute exercise-induced increases in glucose disposal can be offset by increases in glucose appearance rates, thereby altering glucose tolerance [6,15]. Furthermore, it has been suggested that carbohydrate status around the exercise period may be a further mediator of glucose tolerance post-exercise [16]. Therefore, to translate these mechanistic findings for application, an understanding of metabolic responses to food ingestion is needed.

The purpose of this study was to investigate the role of replacing post-exercise carbohydrate on glucose tolerance and insulin sensitivity using an oral glucose tolerance test (OGTT). It was hypothesized that post-exercise replacement of the carbohydrate utilized during 90 min of treadmill running at ~70% $\dot{V}O_{2}$max exercise would impair glucose tolerance and insulin sensitivity the following morning during an OGTT, compared to when the exercise-induced carbohydrate deficit is maintained overnight.

## 2. Materials and Methods

This study was approved by the University of Bath Research Ethics Approval Committee for Health (REACH—EP 15/16 182). Fourteen healthy participants (11 men and 3 women) were recruited for the study (Table 1). Written informed consent was obtained from participants after confirming their understanding of the study design and possible risks. Nine participants (6 men and 3 women) were tested in the United Kingdom and were native to the UK, and five participants (all men) were tested in Taiwan and were native to Taiwan. None of the participants self-reported as smokers. The insulin sensitivity responses to the intervention did not differ between locations (data not shown).

### 2.1. Experimental Design

**Figure 1:**
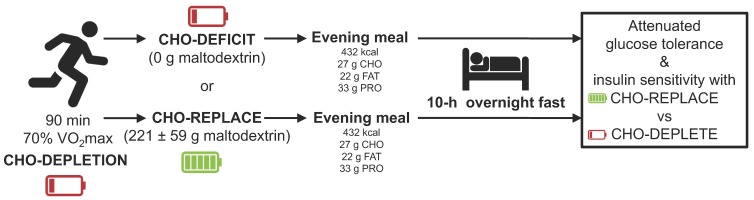
Schematic of the study design. CHO, carbohydrate; FAT, fat, PRO, protein; OGTT, oral glucose tolerance test.

This study was a dual-center (University of Bath and National Chung Hsing University), randomized, double-blind crossover design with two treatment arms. All participants underwent two 2-day experimental trials with a 7-day washout period between trials. On day 1, between 1600–1700 h, participants were asked to run on a treadmill at 70% $\dot{V}O_{2}$max for 90 min. After exercise, subjects were immediately given either a carbohydrate replacement drink (CHO-REPLACE) or placebo drink (CHO-DEFICIT). A low carbohydrate pack-dinner was provided in both trials. The following morning after an overnight fast, participants were asked to perform an oral glucose tolerance test (OGTT; Figure 1). In order to standardize metabolic parameters prior to trials, participants were asked to record their diet 3 days before the first trial and repeated the same diet before the next trial. In addition, they were asked to refrain from smoking and ingesting alcohol- and caffeine-containing beverages for 24 h before the OGTT. All participants reported successful replication of lifestyle prior to trials.

### 2.2. Preliminary Tests

Subjects completed two preliminary tests: a maximal oxygen uptake test ($\dot{V}O_{2}$max) and a familiarization test, at least 1 week before the main trial. The $\dot{V}O_{2}$max test protocol has been described previously [17]. At least 3 days prior to the first main trial, a 60-min familiarization run was performed to accustom participants to an extended period of treadmill-running. The session was also used to re-affirm the appropriate treadmill speed required to achieve an exercise intensity of 70% $\dot{V}O_{2}$max. Accordingly, heart-rate, RPE, and expired air samples were collected and analyzed at 15 min intervals.

### 2.3. Main Trials

Day 1—90-min run: Participants were asked to arrive at the laboratory between 15:30–15:45, and body-mass and stature were recorded. Participants were then fitted with a heart-rate monitor and the 90-min run at 70% $\dot{V}O_{2}$max initiated at ~16:00. After the run, a test drink containing either carbohydrate or placebo was ingested immediately post-exercise and participants were asked to ingest the drink within one hour. Finally, a standardized dinner (432 kcal; 27 g carbohydrate (23 g of which sugars), 22 g fat, 33 g protein) was also provided, to be consumed between 19:30 and 20:00. Participants were then asked to abstain from consuming any further food or drink other than water.

Day 2—Oral Glucose Tolerance Test (OGTT): Participants attended the laboratory between 07:30–07:45, to perform a two-hour OGTT having completed an overnight fast (>10 h) and refrained from further exercise. 

Upon arrival, participants rested for 15-min in a semi-recumbent position with their hand placed in a hot-box set to 55 °C [17], with a subsequent five-minute (baseline) expired air sample collected from a subgroup (*n* = 7). Following this, a cannula was inserted into an antecubital vein of the participant’s forearm and a 5-mL (baseline) blood sample drawn. A 75 g glucose load was then administered orally (113 mL Polycal: Nutricia, UK (mixed with 87 mL water)) and blood samples were collected at 30 min intervals over 2-h. 5-min expired air samples were also collected at 25 to 30, 55 to 60, 85 to 90, and 115 to 120-min from a subgroup (*n* = 7). Since there were some slight differences in protocols between University of Bath and the National Chung Hsing University (namely, Bath protocols included a hot-box for blood sampling and expired breath analysis), the data from both institutions were initially analyzed separately to check that responses were similar. Since both protocols produced similar overall responses for glycaemia, insulinemia, and insulin sensitivity indices, the data were combined for the present manuscript (*n* = 14).

### 2.4. Test Drink

During the CHO-REPLACE trial, a 15% Maltodextrin solution (MyProtein, Cheshire, UK; Batch No.: L626929168) was ingested to precisely replace carbohydrate oxidized during the preceding run. The total amount of carbohydrate utilized during exercise were determined via indirect calorimetry from expired gases collected every 15-min during exercise. The amount of carbohydrate replacement for the CHO-REPLACE trial was 221.4 ± 59.3 g. Conversely, a (0 g carbohydrate) 1.5% artificially-sweetened placebo solution (Truvia, Silver Spoon, Peterborough, UK) was ingested during the CHO-DEFICIT trial.

### 2.5. Blood and Expired Air Samples Collection and Analysis

#### 2.5.1. Blood Sample Collection and Analysis

The arterialized blood samples were obtained via a cannula inserted into antecubital vein of each subject’s forearm [17]. A non-heparinized tube was used to collect 2 mL of blood sample, and it was allowed to stand for 1 h to wait for the blood to coagulate. Another tube containing ethylenediaminetetraacetic acid (EDTA; BD, Oxford, UK) was used to collect 3 mL of blood sample. The collected sample was then centrifuged (Eppendorf 5810, Hamburg, Germany) in 4 °C at 2500 g for 10 min. The extracted serum and plasma samples were stored at −80 °C before later analysis. Serum insulin concentrations were analyzed using enzyme-linked immunosorbent assays (ELISA, Mercodia AB, Uppsala, Sweden), following the manufacturer’s instructions. Minimal detectable concentrations for serum insulin were set at 18 pmol·L^−1^ and intra-plate coefficients of variation were <4.4%. Plasma glucose concentrations were analyzed using a spectrophotometric analyzer (Randox Daytona, Randox Laboratories Ltd., Crumlin, UK). Due to a cannula blockage on one trial for one participant, data for blood-based variables are (*n* = 13).

#### 2.5.2. Expired Gas Samples Collection and Analysis

The Douglas bag method was used to assess substrate metabolism at rest and during exercise. For all samples, participants were provided the mouthpiece before gas collections for a stabilization period. At rest, the stabilization and gas collection periods were each 5 min, whereas during exercise the stabilization and gas collection periods were each 1 min. Samples were collected in 200 L Douglas bags (Hans Rudolph, Kansas City, MO, USA) through falconia tubing (Baxter, Woodhouse and Taylor Ltd., Macclesfield, UK). Expired O_2_ and CO_2_ concentrations were measured in a known volume of each sample, using paramagnetic and infrared transducers, respectively (Mini HF 5200, Servomex Group Ltd., Crowborough, East Sussex, UK). The sensor was calibrated using known concentrations of low (99.998% Nitrogen, 0% O_2_ and CO_2_) and high (balance nitrogen mix, 16.04% O_2_, 5.06% CO_2_) calibration gases (both BOC Industrial Gases, Linde AG, Munich, Germany). Substrate utilization was determined during exercise using the equations of Jeukendrup and Wallis (2005) [18], whilst Frayn’s (1983) [19] equations were used for samples collected at rest as follows (where $\dot{V}O_{2}$ and $\dot{V}CO_{2}$ are expressed in L/min):

(1) $$\mathrm{Fat}\;\mathrm{utilisation}\;\mathrm{at}\;\mathrm{rest}\;\mathrm{and}\;\mathrm{during}\;\mathrm{exercise}\;(g/\min)\;=\;(1.695\;\times \;\dot{V}O_{2})\;-\;(1.701\;\times \;\dot{V}CO_{2})$$

(2) $$\mathrm{Carbohydrate}\;\mathrm{utilisation}\;\mathrm{at}\;\mathrm{rest}\;(g/\min)\;=\;(4.585\;\times \;\dot{V}{\mathrm{CO}}_{2})\;-\;(3.226\;\times \;\dot{V}O_{2})$$

(3) $$\mathrm{Carbohydrate}\;\mathrm{utilisation}\;\mathrm{during}\;\mathrm{exercise}\;(g/\min)\;=\;(4.210\;\times \;\dot{V}{\mathrm{CO}}_{2})\;-\;(2.962\;\times \;\dot{V}O_{2})$$

### 2.6. Sample Size Estimation

The sample size estimation was performed using data on insulin concentrations during steady-state intravenous glucose infusion following exercise training with, or without, carbohydrate and energy replacement. In the absence of carbohydrate replacement, insulin concentrations were ~225 ± 141 pmol·L^−1^, compared to ~345 ± 85 pmol·L^−1^ when the carbohydrate and energy utilized during exercise was replaced. Based on this effect size (*d* = 1.03), 12 participants should provide more than a 90% chance of detecting such an effect with an alpha level of 0.05.

### 2.7. Statistical Analysis

Incremental area under the curve (iAUC; divided by 120 min to provide time average values) and Matsuda Insulin sensitivity index (Matsuda index; [18]) were calculated from plasma glucose and serum insulin data using Microsoft Excel (Version 15.26, Microsoft, Redmond, WA, USA). The updated homeostasis assessment model of insulin resistance (HOMA2-IR; [19]) was calculated using freely available online software (https://www.dtu.ox.ac.uk/homacalculator/). Statistical analyses were performed using GraphPad Prism v7 (GraphPad Software, San Diego, CA, USA). Differences between trials in time-dependent variables (glucose and insulin concentrations, and carbohydrate and fat oxidation rates) were analyzed by a two-way ANOVA with repeated measures. For non-time dependent variables, paired *t*-tests were applied. A *p*-value of ≤0.05 was considered statistically significant. Data are presented in the body of the text as mean ± SD, whereas error bars on figures are confidence intervals normalized to remove between subject variance, consistent with this within-subject design [20]. With this approach, any error bars that do not overlap the mean of their respective comparison can be considered to have a significance level of <0.05.

## 3. Results

### 3.1. Indirect Calorimetry during the Treadmill Run

The mean rates of oxygen consumption and carbon dioxide production were 2.65 ± 0.43 L·min^−1^ and 2.65 ± 0.48 L·min^−1^, respectively. This resulted in a respiratory exchange ratio of 1.00 ± 0.12 ($\dot{V}O_{2}$:$\dot{V}{\mathrm{CO}}_{2}$). Based on data collected at Bath, the total amount of carbohydrate oxidized during the run in CHO-DEPLETE was 205 ± 58 g vs. 220 ± 52 g in the CHO-REPLACE trial.

### 3.2. Glycemia, Insulinemia and Insulin Sensitivity

**Figure 2:**
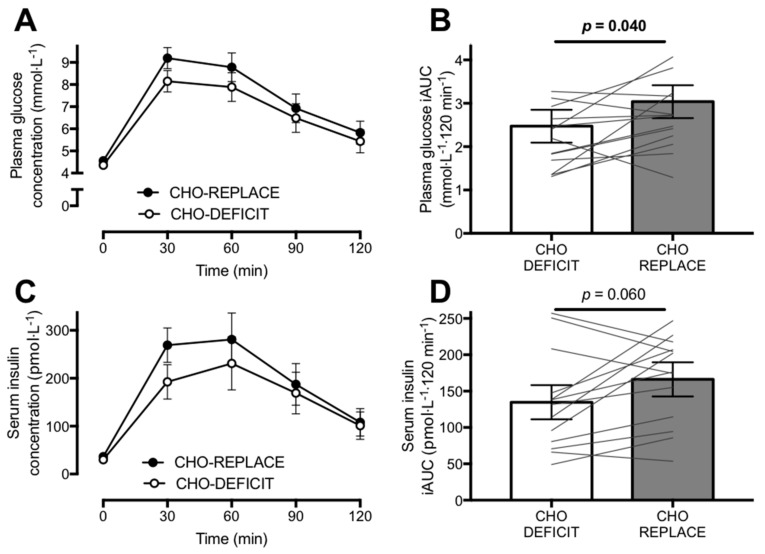
Postprandial glycaemia (**A**,**B**) and insulinemia (**C**,**D**) expressed as absolute concentrations (**A**,**C**) or as the incremental time-averaged area under the curve (iAUC; **B**,**D**) during the oral glucose tolerance test conducted ~16 h after exercise with either carbohydrate replacement (CHO-REPLACE) or a maintenance of the exercise-induced carbohydrate deficit (CHO-DEFICIT). *n* = 13. Data are means ± normalized 95% CI.

Pre-OGTT glucose concentrations were 4.56 ± 0.55 mmol·L^−1^ during the CHO-REPLACE trial and 4.36 ± 0.51 mmol·L^−1^ during the CHO-DEFICIT trial (*p* = 0.149). Following ingestion of the OGTT, plasma glucose concentrations rose to a greater extent in CHO-REPLACE versus CHO-DEFICIT (Figure 2A,B; *p* = 0.040), whereby peak glucose concentrations were 9.74 ± 1.22 mmol·L^−1^ on the CHO-REPLACE and 8.33 ± 1.76 mmol·L^−1^ on the CHO-DEFICIT trial. Repeated measures ANOVA revealed main effects of time (*p* < 0.001) and treatment (*p* = 0.007), but no time–treatment interaction effect (*p* = 0.170).

Pre-OGTT insulin concentrations were higher during the CHO-REPLACE trial compared to the CHO-DEFICIT trial (36 ± 31 pmol·L^−1^ compared to 30 ± 20 pmol·L^−1^, respectively *p* = 0.023). Following ingestion of the OGTT, the increase in serum insulin concentrations was greater with CHO-REPLACE versus CHO-DEFICIT (Figure 2C,D), whereby peak insulin concentrations were 337 ± 107 pmol·L^−1^ during CHO-REPLACE, compared to 260 ± 101 pmol·L^−1^ during CHO-DEFICIT (*p* = 0.012). Repeated measures ANOVA revealed main effects of time (*p* < 0.001) and treatment (*p* = 0.028), but no time—treatment interaction effect (*p* = 0.158).

**Figure 3:**
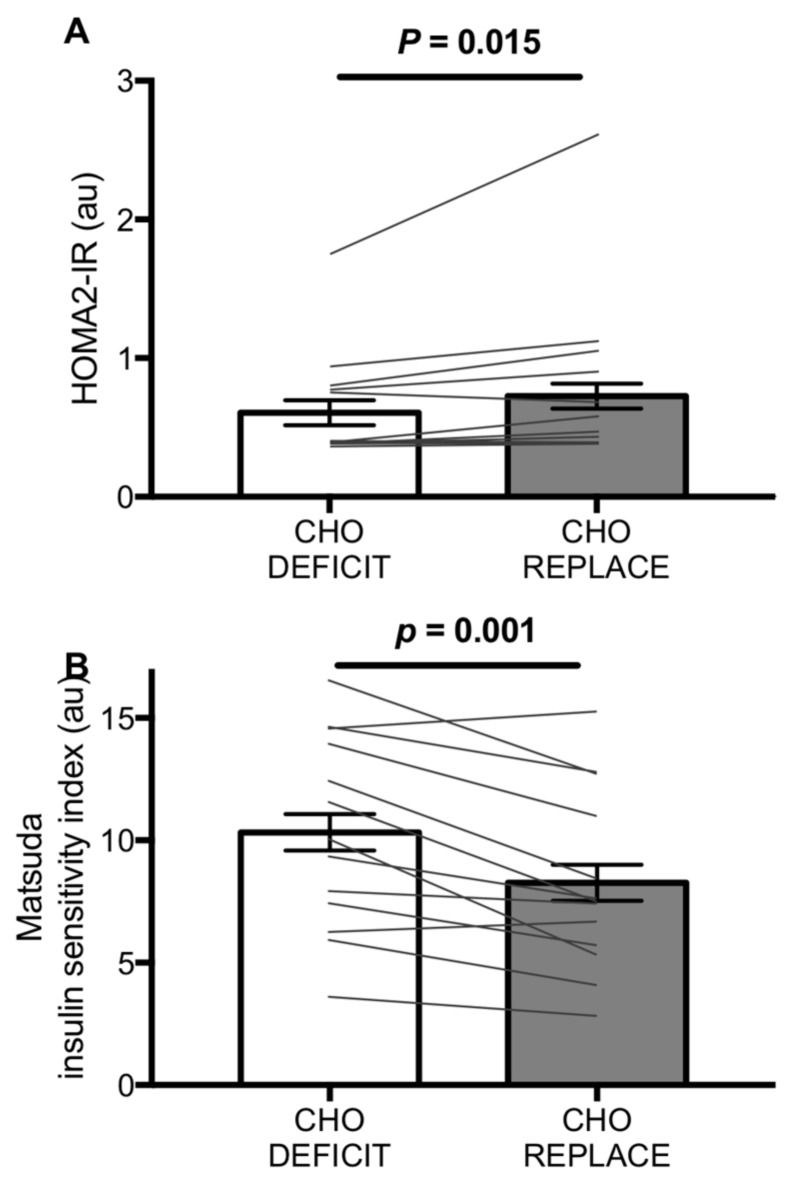
Homeostasis model of insulin resistance (HOMA2-IR) (**A**) and the Matsuda insulin sensitivity index (**B**) during the oral glucose tolerance test conducted ~16 h after exercise with either carbohydrate replacement (CHO-REPLACE) or a maintenance of the exercise-induced carbohydrate deficit (CHO-DEFICIT). *n* = 13. Data are means ± normalized 95% CI.

The HOMA2-IR was ~16% higher with CHO-REPLACE versus CHO-DEFICIT (Figure 3A; *p* = 0.015), whereas the Matsuda insulin sensitivity index was ~25% lower with CHO-REPLACE vs. CHO-DEFICIT (Figure 3B; *p* = 0.001).

### 3.3. Whole-Body Substrate Utilisation

**Figure 4:**
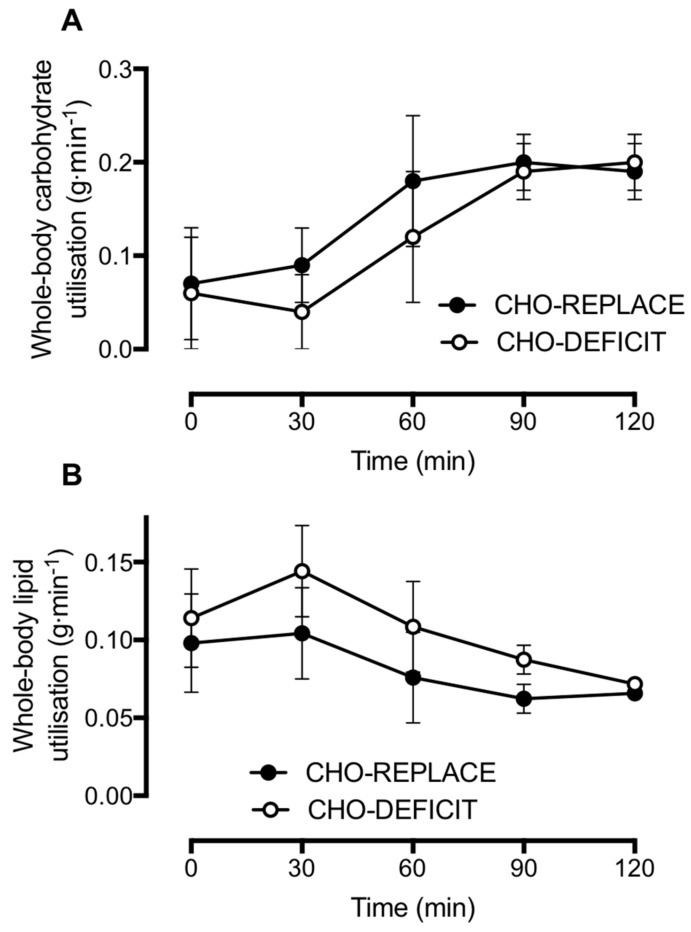
Whole-body carbohydrate (**A**) and lipid utilization (**B**) during an oral glucose tolerance test conducted ~16 h after exercise with either carbohydrate replacement (CHO-REPLACE) or a maintenance of the exercise-induced carbohydrate deficit (CHO-DEFICIT). *n* = 7. Data are means ± normalized 95% CI.

Pre-OGTT, whole-body carbohydrate utilization was 0.08 ± 0.05 g·min^−1^ during the CHO-REPLACE trial and 0.06 ± 0.05 g·min^−1^ during the CHO-DEFICIT trial (*p* = 0.639). Following ingestion of the OGTT, carbohydrate utilization increased ~2-fold in both trials (Figure 4A; time effect: *p* < 0.001), with no differences between trials (treatment effect: *p* = 0.378; time–treatment interaction effect: *p* = 0.099).

Pre-OGTT, whole-body lipid utilization was 0.10 ± 0.03 g·min^−1^ during the CHO-REPLACE trial and 0.11 ± 0.03 g·min^−1^ during the CHO-DEFICIT trial (*p* = 0.350). Following ingestion of the OGTT, lipid utilization was suppressed in both trials (Figure 4B; time effect: *p* < 0.001), but to a greater extent in CHO-REPLACE vs. CHO-DEFICIT (Figure 4B; treatment effect: *p* = 0.033; time–treatment interaction effect: *p* = 0.048).

## 4. Discussion

The present study demonstrates that replacement of the carbohydrate utilized during a single bout of exercise impairs both insulin sensitivity and glucose tolerance by ~20–25% the following morning, relative to when the exercise-induced carbohydrate deficit is maintained. Importantly, these changes were most clearly apparent in the postprandial state. Furthermore, postprandial fat oxidation was suppressed by post-exercise replacement of carbohydrate use.

Previous work has demonstrated that, whilst exercise is a potent method of stimulating muscle glucose uptake and insulin sensitivity, the carbohydrate deficit induced by exercise is key factor that mediates these responses. However, previous work has primarily used intravenous methods of assessing insulin sensitivity and/or action, which do not necessarily translate into the tolerance of ingested nutrients.

It has been suggested that the degree of whole-body carbohydrate depletion is a key mediator of exercise induced-increases in insulin action. Indeed, a positive relationship has been reported between post-exercise carbohydrate depletion and the change in insulin action assessed during intravenous glucose infusion, whereby a carbohydrate deficit of greater than 90 g was associated with an increase in insulin action [21]. In the present study, the mean carbohydrate deficit was 221 ± 59 g; all participants had a carbohydrate deficit of at least 99 g and there was a clear ~25% increase in insulin sensitivity as assessed by the Matsuda index, which was apparent in 12 of 14 individuals. Furthermore, whilst we did not have an energy-matched, low-carbohydrate condition to isolate the effect of carbohydrate versus energy-replacement, it has previously been demonstrated that re-feeding fat post-exercise does not influence glucose tolerance or insulin sensitivity the following morning [22]. Therefore, our data extend those findings demonstrating that post-exercise carbohydrate re-feeding does influence glucose tolerance and insulin sensitivity the following morning. Taken together, the evidence suggests that whole-body carbohydrate depletion induced by exercise is a key mediator of the enhanced insulin sensitivity and glucose control induced by exercise.

We report that both HOMA2-IR and the Matsuda insulin sensitivity index indicated an impairment in insulin sensitivity with post-exercise carbohydrate replacement, by ~16% and ~25%, respectively. Fasting concentrations of insulin and glucose primarily reflect changes in hepatic insulin sensitivity, whereas at postprandial concentrations, hepatic glucose production is negligible [18,23]. On this basis, it has been suggested that HOMA2-IR is primarily reflective of hepatic insulin sensitivity, whereas the Matsuda insulin sensitivity index is more heavily influenced by peripheral insulin sensitivity [24]. The finding that postprandial metabolic responses are more clearly altered than fasted measures by post-exercise carbohydrate replacement suggests that peripheral insulin sensitivity was more heavily influenced by replacement of carbohydrate compared to hepatic insulin sensitivity. It should be acknowledged that the present data does not allow for interpretation of insulin secretion to be assessed. Therefore, the reduction in glucose tolerance with carbohydrate replacement compared to the maintenance of the carbohydrate deficit could be due, in part, to an inability of the pancreas to secrete sufficient insulin to compensate for the change in insulin sensitivity. Furthermore, it has previously been shown that the timing of carbohydrate re-feeding post-exercise can alter insulin action the following day [11]. Delaying the re-feeding of carbohydrate by 3 h results in lower insulin action compared to immediate post-exercise carbohydrate refeeding. Therefore, the immediate re-feeding in the present study may result in a lower-bound estimate of the impairment in insulin sensitivity with carbohydrate replacement in the hours following exercise.

During a prolonged bout (90 min) of moderate-to-high intensity exercise (70% $\dot{V}O_{2}$peak), both muscle and liver glycogen concentrations can be expected to be depleted by ~60% [25,26,27]. In the absence of meaningful quantities of carbohydrate or glycogenic amino acid ingestion, only negligible net quantities of muscle and liver glycogen will be synthesized. Therefore, prior to the OGTT we can be confident that both muscle and liver glycogen stores would have been depleted with the carbohydrate restriction trial, compared to the carbohydrate replacement trial. Accordignly, the observed suppression of insulin sensitivity with replacement of carbohydrate is likely to represent depletion of all major glycogen stores. This is important, since hepatic and muscle insulin sensitivity appear to respond differentially to carbohydrate status during acute (3-d) overfeeding [28].

Increased insulin sensitivity after exercise does not always translate into changes in glucose control after the ingestion of nutrients. Post-exercise increases in glucose disposal can be offset by changes in the rate of appearance of glucose from both endogenous and exogenous sources [6], leading to either no change or even a worsening of glucose tolerance after a single bout of exercise [7]. This highlights the importance of complementing mechanistic studies of insulin sensitivity that involve intravenous infusion methods, with oral ingestion of nutrients. In the present study, we employed an oral glucose tolerance test and demonstrated that restoring carbohydrate balance via post-exercise feeding increases postprandial glycaemia compared to a maintenance of the exercise-induced carbohydrate deficit.

We also observed a difference in postprandial substrate metabolism, whereby whole-body lipid utilization was suppressed when the carbohydrate deficit of prior exercise had been replaced. This suppression of postprandial lipid utilization is consistent with findings from others in which the energy utilized by exercise was replaced [12] and further highlights the role of carbohydrate balance in the regulation of whole-body lipid utilization. Furthermore, the greater suppression of postprandial lipid utilization with carbohydrate replacement is consistent with the high fasting and postprandial insulinemia that we observed, since insulin is a potent inhibitor of adipose tissue lipolysis in vivo [29].

The present study design does not allow for inferences to be drawn about the effects of exercise on insulin sensitivity and glycemic control, since there was no non-exercise trial. Therefore, the carbohydrate replacement in this study could be either: (1) partly attenuating the effects of exercise; (2) completely reversing the effects of exercise; or (3) superseding the effects of exercise. However, the effects of exercise on glycemia and insulin sensitivity are well-characterized, and the aim of the present investigation was specifically to establish the degree to which the carbohydrate deficit of exercise alters glycemia and insulin sensitivity. By comparing the carbohydrate replacement trial with the maintenance of the exercise-induced carbohydrate deficit, we are able to establish the extent to which the whole-body carbohydrate deficit alters postprandial glycaemia and insulin sensitivity. Furthermore, the findings of this study will need further work to provide greater generalizability and to further characterize the underlying mechanisms. In order to be able to generalize the findings to people at risk of metabolic disease, this work should be repeated in overweight/obese people, and at lower exercise intensities. Some disease states and lower exercise intensities would reduce the reliance on glycogen use during exercise, thereby altering the nutrition interaction with exercise. Furthermore, to firmly establish the underlying mechanisms, isotopic tracers and euglycemic hyperinsulinemic clamps could establish rates of appearance and disappearance of glucose, and peripheral insulin sensitivity, respectively. Nonetheless, the present study provides the first evidence, as proof of principle, that replacing the carbohydrate deficit induced by exercise has the capacity to reduce postprandial glycemic control and insulin sensitivity the following morning.

## 5. Conclusions

This study is the first to show that feeding carbohydrate to replace that utilized during exercise can reduce insulin sensitivity and glucose tolerance the next morning in healthy adults, when compared to a preservation of the exercise-induced carbohydrate deficit. Furthermore, carbohydrate replacement suppresses subsequent postprandial fat utilization. The mechanism through which exercise improves insulin sensitivity and glucose control is therefore (at least partly) dependent on carbohydrate availability, and so the day-to-day metabolic health benefits of exercise might be best attained by maintaining a carbohydrate deficit overnight.

## Figures and Tables

**Table 1:** Summary of participant characteristics, *n* = 14.

	Mean ± SD
Age (years)	24 ± 5
Height (m)	1.76 ± 0.06
Mass (kg)	71.1 ± 9.0
BMI (kg/m^2^)	23.6 ± 4.5
$\dot{V}O_{2}$max (mL·kg^−1^·min^−1^)	56 ± 10
